# Study Protocol for a Cluster, Randomized, Controlled Community Effectiveness Trial of the Early Start Denver Model (ESDM) Compared to Community Early Behavioral Intervention (EBI) in Community Programs serving Young Autistic Children: Partnering for Autism: Learning more to improve Services (PALMS)

**DOI:** 10.1186/s40359-024-02020-0

**Published:** 2024-09-28

**Authors:** Aubyn C. Stahmer, Sarah Dufek, Sally J. Rogers, Ana-Maria Iosif

**Affiliations:** 1 UC Davis Health, MIND Institute, University of California, 2825 50th St., Sacramento, CA 95819 USA; 2 UC Davis Health, Department of Public Health Sciences, University of California, One Shields Ave., Davis, CA 95616 USA

**Keywords:** Autism, Early intervention, Early start Denver model, NDBI, Community implementation

## Abstract

**Background:**

The rising number of children identified with autism has led to exponential growth in for-profit applied behavior analysis (ABA) agencies and the use of highly structured approaches that may not be developmentally appropriate for young children. Multiple clinical trials support naturalistic developmental behavior interventions (NDBIs) that integrate ABA and developmental science and are considered best practices for young autistic children. The Early Start Denver Model (ESDM) is a comprehensive NDBI shown to improve social communication outcomes for young autistic children in several controlled efficacy studies. However, effectiveness data regarding NDBI use in community-based agencies are limited.

**Methods:**

This study uses a community-partnered approach to test the effectiveness of ESDM compared to usual early behavioral intervention (EBI) for improving social communication and language in autistic children served by community agencies. This is a hybrid type 1 cluster-randomized controlled trial with 2 conditions: ESDM and EBI. In the intervention group, supervising providers will receive training in ESDM; in the control group, they will continue EBI as usual. We will enroll and randomize 100 supervisors (50 ESDM, 50 EBI) by region. Each supervisor enrolls 3 families of autistic children under age 5 (n = 300) and accompanying behavior technicians (n = 200). The primary outcome is child language and social communication at 6 and 12 months. Secondary outcomes include child adaptive behavior, caregiver use of ESDM strategies, and provider intervention fidelity. Child social motivation and caregiver fidelity will be tested as mediating variables. ESDM implementation determinants will be explored using mixed methods.

**Discussion:**

This study will contribute novel knowledge on ESDM effectiveness, the variables that mediate and moderate child outcomes, and engagement of its mechanisms in community use. We expect results from this trial to increase community availability of this model and access to high-quality intervention for young autistic children, especially those who depend on publicly funded intervention services. Understanding implementation determinants will aid scale-up of effective models within communities.

**Trail registration:**

Clinicaltrials.gov identifier number NCT06005285. Registered on August 21, 2023.

**Protocol version:**

Issue date 6 August 2024; Protocol amendment number: 02.

## Background

### Autism as an ongoing significant public health concern

Autism Spectrum Disorder (autism) continues to be one of the most common forms of neurodevelopmental disability worldwide, with US estimates of 1 in 36 children [1]. Autism presents a significant public health challenge in that the average per capita lifetime costs of challenges associated with an autism diagnosis in the US exceeds $3 million. The societal costs are over $7 trillion and are projected to rise to $14 trillion by 2029 [2, 3]. High-quality, evidence-based early intervention has the potential to improve child outcomes by reducing intellectual impairment and improving social communication and language skills [4–7]. In addition, research suggests that the cost of early evidence-based intervention may be offset by reduced costs of special education and other intervention across the lifespan [2, 8, 9]. The most frequently used early intervention for autistic children under age 5 is behavioral therapy [10]. A recent survey reports approximately 40% of autistic children in the US receive intensive behavior therapy (17–67% depending upon region) [11]. However, current data come primarily from controlled efficacy studies with strict inclusion criteria, highly trained providers and limited sample diversity [12].

Because of increasing demand due to rising prevalence, consumer knowledge, and improved insurance coverage, the US has seen a proliferation in the number of for-profit autism community-based agencies (CBAs) offering intervention. Since the Affordable Care Act (2010), 47 states have mandates for insurance funding of autism interventions based on Applied Behavior Analysis (ABA). For a variety of reasons, including initial studies from several decades ago, structured interventions based on ABA are most often used in CBAs [13]. CBAs are estimated to serve over 50,000 autistic people and generate $1.07 billion in revenues annually. They serve many historically underrepresented autistic children, including those living in low-income communities. However, the fast growth in number of CBAs belies the lack of effectiveness research for their services. This lack of the necessary evidence-base to support current community practice raises serious public health concerns about the cost, effectiveness, and quality of community early autism intervention. This is especially true for children of color [14].

### Need for improvement in community-based early intervention services for autistic children

Systematic reviews and meta-analyses of randomized efficacy trials find positive effects of both highly-structured, ABA-based early interventions, and naturalistic developmental behavioral interventions (NDBIs) on developmental outcomes for young children with autism [15–17]. Discrete Trial Teaching (DTT), a highly-structured intervention based on ABA principles, was one of the first identified early interventions for autism [18]. DTT has a clear, structured curriculum and highly scripted teaching strategies. It is adult-directed and uses massed trial-based learning and external motivation to teach skills across domains. DTT is the primary comprehensive strategy taught in a majority of BCBA and technician training programs and therefore represents the primary strategy provided by CBAs [19]. Most effectiveness studies for the DTT model are either case–control studies or quasi-experimental with a few randomized controlled design studies [20–23]. Structured ABA programs often demonstrate better outcomes for children than eclectic models or waitlist controls; however, results are inconsistent [24, 25] and effect sizes are consistently smaller than in efficacy trials [26–28].

In the past 25 years since the initial non-randomized DTT efficacy trial [18], intervention sciences has evolved to bring developmental science into early intervention via the NDBIs [29]. NDBIs combine developmental science with ABA principles to include developmentally appropriate learning targets and teaching strategies, including those that integrate child learning into daily activities to build well-generalized child learning. Additionally, these developmentally appropriate practices respect young children’s interests, choices, and initiative, and focus on children’s own motivations to support child-directed learning. The NDBI evidence base includes multiple randomized trials testing NDBIs [30–33], which are established best practice for young autistic children [34], supported by systematic reviews and meta-analyses reporting positive child outcomes [7, 35, 36].

In addition to being effective, NDBIs facilitate inclusion through their use of typical, developmentally appropriate practices [37, 38]. Additionally, autistic adults have raised concerns regarding the ethics of traditional ABA approaches that focus on compliance, suppression of characteristically autistic behaviors, and “curing” autism, fearing that such approaches pathologize autism and may cause harm to autistic people [39]. While views vary widely, many neurodiversity advocates support person-centered, respectful intervention focusing on skill building and improving quality of life [40]. NDBIs use a strengths-based approach focused on child choices and preferences to support child learning, especially in social communication and language. NDBIs have potential to better align early intervention with the goals of autistic individuals [41]. However, NDBIs have a paucity of effectiveness trials in CBAs with the few existing studies flawed by lack of experimental designs [42], a focus on school settings [43–45], or low-intensity, parent-implemented studies [46, 47].

Currently, CBAs rarely implement NDBI models, instead using highly structured DTT strategies not developmentally appropriate for young children, [48, 49] due in part to lack of knowledge and quality training. A recent survey of behavior therapists found that few recognize or understand how to use NDBIs in practice [19]. Given the strengths of NDBI, the large numbers of CBAs serving young autistic children, and their lack of NDBI knowledge, there is a clear need for effectiveness testing with diverse children in community care to determine if NDBIs support child learning and progress and family use and satisfaction.

### Need for effectiveness testing of NDBI to meet this need

One NDBI developed for autistic children under age 5 is the Early Start Denver Model (ESDM) [29]. ESDM is a comprehensive model that aims to increase children’s social motivation and social learning opportunities, decrease their developmental delays, and enhance social communication. ESDM uses a data-based approach and empirically supported ABA and developmental teaching practices embedded in everyday activities. ESDM integrates ABA with developmental, relationship- and play-based practices to create an integrated approach that is individualized while also being standardized and manualized. ESDM fits within the seven dimensions that define ABA practice [50–52] and meets the criteria of the Professional and Ethical *Compliance Code for Behavior Analysts* [53] and additional parameters in the *ABA Treatment of ASD Practice Guidelines* [54].

ESDM is one of the very few comprehensive early interventions validated and replicated in multiple, randomized trials [30, 33, 55]. A recent meta-analysis found significant effects of ESDM on cognition and language compared to usual care [4]. ESDM is effective for autistic children across a wide range of learning styles and abilities and is flexible enough to be used in many contexts by different types of providers and caregivers. While the evidence-base is strong, in these studies ESDM has been delivered by highly trained providers at university-based research sites. Exclusion criteria in some trials eliminated participants based on severe caregiver mental health conditions, geographic location, limited English, and child characteristics (e.g., IQ < 35, genetic comorbidities) [56].

To date, there have been no effectiveness trials of the comprehensive ESDM protocol in community settings. However, a recent review found that ESDM has more evidence to support its use with participants from culturally and linguistically diverse backgrounds than any other NDBI [57]. Two feasibility studies suggest community effectiveness when treatment is provided by ESDM-certified staff [58, 59]. A recent randomized feasibility trial of an adapted model (Community ESDM; C-ESDM) in low resource settings demonstrated the feasibility of training community providers to coach caregivers in ESDM and found significant skill gains in provider and caregiver use of ESDM strategies. However, perhaps due to very low intensity, there were no significant differences in the amount of child gain between groups [46]. A recent randomized trial with a broad inclusion criteria that examined the use of C-ESDM in 16 community agencies in Canada involving 49 children found that families receiving C-ESDM reported higher quality of life, intervention satisfaction, and self-efficacy than the comparison group. Children in the C-ESDM group made greater gains in receptive language and faster gains in joint attention and language with greater effect sizes than the comparison group [56]. These feasibility studies show the promise of ESDM for the community and highlight the need for a full-scale effectiveness trial.

### Advancing the science of intervention mechanisms through an ESDM effectiveness trial

Child social motivation and Systematic Caregiver Coaching are the main variables in the hypothesized model of change underlying ESDM [60]. Social motivation theory has been used to explain an underlying mechanism of social communication challenges in autism [61, 62]. Dawson posits that a biological disruption of social motivation results in decreased social attention and social learning beginning in the first year of life, potentially contributing to the developmental delays in learning, social communication, social cognition and social interests observed in autism. Studies have identified early differences in social motivation between autistic and neurotypical individuals in behavioral manifestation, physiology and neurobiology from infancy on [63–65]. A recent meta-analysis showed that, in over 6000 participants, autistic individuals showed reduced social orienting compared to neurotypical peers [66]. Limited social orienting has a negative impact on language learning, [67–70] and early social motivation in autistic toddlers predicts language 2 years later [71]. These studies have led researchers to identify social motivation as a potential mediator of response to early intervention and an important mechanism to target in early treatment [60].

Social motivation has thus been suggested as the underlying mechanism of change in ESDM [72]. Given the plasticity of brain development in the first few years of life and its proclivity for social communication and language learning in this period, ESDM was developed to differentially support social attention, engagement, communication and social motivation to very young autistic children by pairing developmentally appropriate learning with children’s preferred people, activities, and materials. Caregivers learn to use different strategies that better support learning and social engagement for their autistic children. Thus, the ESDM intervention approach heightens the value of social engagement in a way that fits the child’s learning style and supports child social attention for learning. These additional social learning experiences are thought to stimulate further neural development and connectivity resulting in accelerated learning rates overall and improved growth and development of early social communication. This study represents the largest examination of ESDM or any other NDBI yet conducted and will provide additional data about whether ESDM activates the proposed mechanism – social motivation – to improve social communication and language outcomes in autistic children.

ESDM’s strong emphasis on systematic caregiver coaching facilitates child outcomes by allowing caregivers, with whom children may have the highest social motivation, to embed ESDM strategies throughout daily family routines. Coaching caregivers in strategies that increase social motivation can increase learning opportunities and child engagement beyond the treatment session. Inclusion of caregivers in intervention is best practice [73], and fidelity to intervention practices predicts child outcomes in programs with both provider- and caregiver-implemented components [74, 75]. CBA programs typically include some coaching related to behavior concerns rather than teaching intervention strategies [76]. Therefore, having a systematic method of teaching caregivers to use ESDM strategies may increase access to intervention further activating the social motivation mechanism and improve child outcomes The importance of caregiver coaching is further supported by the positive relationship between caregiver fidelity and child outcomes across multiple studies [74, 77]. Therefore, we aim to understand the mediating role of caregiver NDBI fidelity and child outcomes across both groups in this diverse community sample.

### Using implementation science to maximize efficiency and relevance of the ESDM trial

**Fig. 1:**
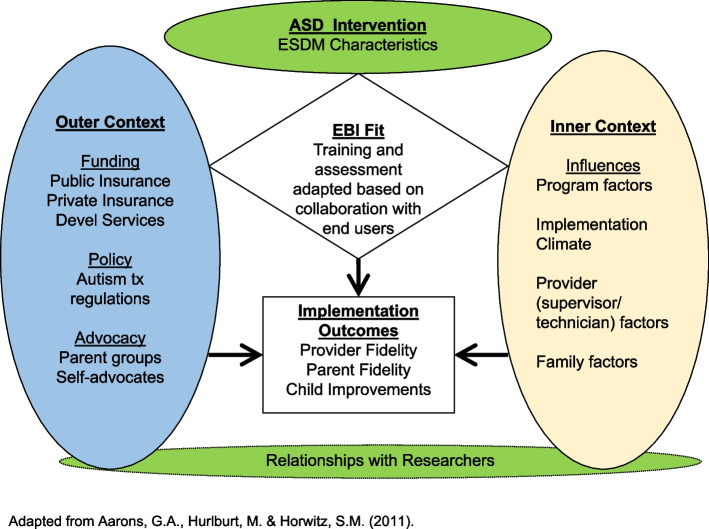
Applying the Exploration, Preparation, Implementation, Sustainment (EPIS) Conceptual Model of Implementation to ASD EBI

If ESDM is effective for autistic children in the community, and acceptable to community providers and caregivers, the next target should be scaling up for broader implementation. This study will use the Exploration, Adoption/Preparation, Implementation, Sustainability (EPIS) framework [78], a multi-level, multi-phase, process and determinant framework to collect preliminary implementation data. This framework both describes the process of translating research into practice and allows for identification of factors that influence implementation outcomes. The EPIS framework is relevant for understanding the process and determinants of service implementation in public service systems for young autistic children (see Fig. 1). EPIS specifies both the critical role of intervention characteristics and the inner and outer context factors on implementation, while attention to client diversity and potential needs for adaptations across levels. Thus, using an implementation science framework can reduce inequities in healthcare delivery [79].

The current project uses a hybrid type 1 randomized controlled design to examine ESDM effectiveness and to gather data on implementation determinants. This study will test the effectiveness of ESDM for improving social communication and language (primary), adaptive behavior, goal progress and quality of life (secondary) outcomes in a diverse community sample of autistic children. Our research question include: (1) Compared to usual EBI, do children in the ESDM condition demonstrate significantly increased growth rates in social communication and language? (2) Do caregivers in the ESDM condition have greater increases in use of general NDBI strategies and greater caregiver competence than those in EBI? (3) Does ESDM engage the treatment mechanisms of child social motivation and caregiver fidelity within both treatment groups? (4) Do variable such as caregiver education, child race/ethnicity and provider adherence to ESDM fidelity moderate child progress in both groups? We will use the EPIS framework to gather data on ESDM Implementation outcomes including acceptability, feasibility, appropriateness (including for children), cultural responsivity, CBA provider ESDM fidelity, and caregiver engagement.

## Methods

### Design & Randomization

**Fig. 2:**
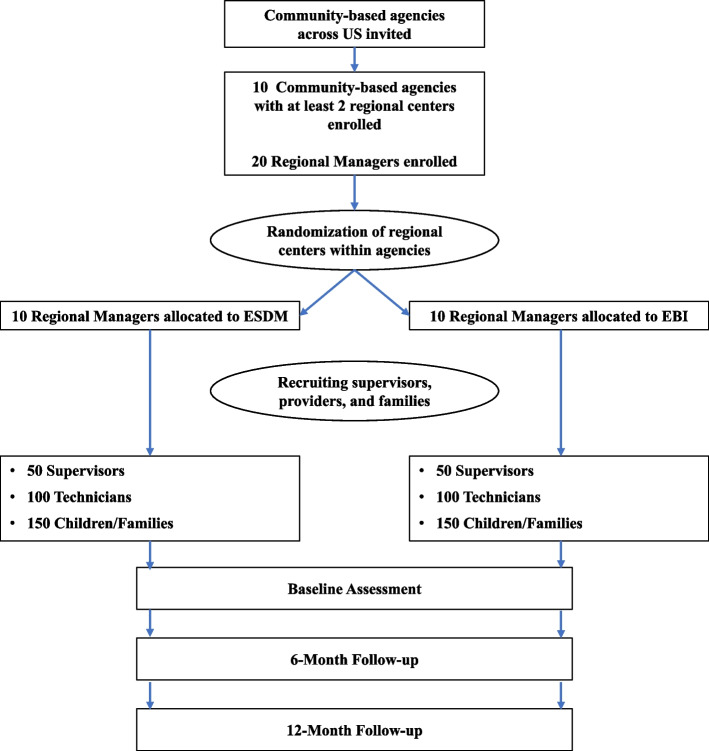
CONSORT flowchart for recruitment of community-based agencies and participants (projected)

This study uses a parallel 2-arm, hybrid type 1 (effectiveness/implementation) cluster randomized controlled trial design. The two arms are ESDM and EBI. We will recruit a multilevel sample (Fig. 2), including 20 CBAs, 20 regional managers, 100 Regional Teams (program supervisor and technicians: average of 1 supervisor and 2 technicians per team) and 300 child/caregiver dyads (2–4 per team). Regional managers from participating regions will complete baseline and follow-up surveys and semi-structured interviews. We will recruit as many supervisors per region as possible with an expected mean of 5 per region. We will recruit as many technicians per region as possible, with replacement to account for high turnover, with an expected mean of 2 per supervisor.

CBAs throughout the US will be recruited, through emails, presentations at conferences and social media, to increase diversity and generalizability. Interested CBA leaders will be invited to meet with the study team. Those CBAs expressing interest and meeting inclusion criteria (see below) will be enrolled in the study. The randomization unit is the region. Within each CBA, regions will be randomized to either receive training in ESDM or continue usual early behavioral intervention (EBI). We chose to randomize at the region level to prevent potential contamination across providers and children, as our community partners indicated that children often receive treatment from multiple providers within a region. Using covariate constrained randomization, regions will be randomized so each CBA is represented in both ESDM and EBI. The variables considered in the constrained randomization include insurance mix (proportion of clients with Medicaid < 0.5 or ≥ 0.5) and size (number of autistic children under age 5 < 20 or ≥ 20). The study statistician will generate the randomization scheme before the enrollment of the first CBA and reveal the random assignments to the appropriate members of the study team. Members of the study team involved in assessments will remain unaware of the intervention assignment.

A cascading recruitment strategy will be used to first recruit agencies and then supervisors within participating regions. Supervisors will then recruit children and families and the technicians working with those children. Recruitment at each level will be facilitated through videos and handouts explaining the study and the processes. The research team will present to interested supervisors at team meetings. Supervisors will receive a handout and link to a video to share with technicians and families. Interested technicians and families will set up a time to talk with the research team about the study to determine interest.

This study was approved by the institutional review board at the University of California, Davis, protocol number 203076–2. This study is funded by the National Institute of Mental Health (NIMH; R01MH131703 and supported by the MIND Institute Intellectual and Developmental Disabilities Research Center (IDDRC) funded by the National Institute of Child Health and Human Development (NICHD; P50 HD103526).

### Participants

#### Community-based agencies

Eligibility criteria for autism CBA include: (a) serve at least 10 children on the autism spectrum under age 5 annually; (2) have at least 2 regions that can be randomized, and (3) accept Medicaid or equivalent payment (e.g., funding for low-income families through public service systems).

#### Supervisor participants

Supervisors will be recruited from enrolled agencies. To be eligible, supervisors must plan to be employed by the agency for at least 12 months, supervise programs for autistic children under age 5, supervise at least two technicians, and have not had previous ESDM training.

#### Technician participants

Technicians supervised by a participating supervisor and working with an enrolled child/family will be recruited. Inclusion criteria include planning to be employed at the agency for at least 12 months and have not had previous ESDM training.

#### Child / family participants

All child clients meeting eligibility criteria with a participating supervisor will be referred to the study and randomly selected for recruitment by the research team, with an expected average of 3 (range 2–4) per supervisor. Inclusion criteria include being under age 4 at program entry, having a current diagnosis of autism or being served by the agency due to high likelihood of autism. The family must speak English or Spanish and plan to receive intervention for at least 7 months. We will confirm autism diagnosis through record review. Payors typically require that children enter treatment with a cognitive assessment and an Autism Diagnostic Observation Scale (ADOS-2) [80]. For children under three who do not have a confirmed autism diagnosis we will complete the Telemedicine-based Autism Evaluation Tool for Toddlers and Young Children (TELE-ASD-PEDS) [81] found to be feasible and effective at assessing autism over telehealth [82, 83].

### Clinical intervention and community training

#### Clinical intervention

Treatment will be conducted in the community context of the CBAs serving autistic children under age 5. They accept payment through insurance (public or private) or contracts with public agencies (e.g., Department of Developmental Services). CBA structure typically involves treatment teams that include a supervising clinician with a Master’s degree and credentials such as a BCBA, and 2–10 technicians. Supervisors conduct assessments, develop, and monitor treatment programs, provide caregiver coaching, and train and supervise technicians. Technicians have approximately 40 h of training in autism treatment and standardized supervision based on payor and board requirements; they conduct 1:1 intervention sessions with the child. Treatment intensity varies based on child need, family preference and payor requirements; however, most agencies provide 10–30 h per child per week of intervention which has been shown to be effective for this age group [55].

#### Early Start Denver Model (ESDM)

The Early Start Denver Model [30, 72] focuses on teaching inside children’s play and care activities, carried out within a joint activity structure [84]. Adults follow children’s leads into activities, embed teaching objectives inside the activity, use the play or child’s activity goal as the reward, and build targeted skills by applying ESDM teaching strategies from developmental science and ABA principles. ESDM uses a developmental curriculum that defines the skills to be taught in each area of development based on each child’s strengths and needs. Core features of ESDM include child preferred materials and activities, use of both developmental strategies and naturalistic ABA strategies, a focus on teaching developmentally appropriate, well-generalized functional skills, caregiver involvement, and a focus on positive social interactions embedded within everyday activities. ESDM uses decision trees to determine when and how to vary the primary, child-centered teaching practices to assure child progress. ESDM Fidelity Tools measure the quality of implementation (see below). Providers in regions randomized to ESDM will receive ESDM training as described below.

#### Usual early behavioral intervention (EBI)

Treatment as usual will vary based on the agency. However, a majority of CBAs use Discrete Trial Teaching (DTT) based on the Lovaas model [18]. DTT involves 10 components described in numerous research publications [85]: capturing child physical and visual attention, adult presentation of the stimuli and instruction (antecedent), child behavior, adult reinforcement, correction procedures, 3–5 s interstimulus interval between trials, behavior-specific praise, and data recording. The use of DTT and NDBI strategies will be measured across both groups (see below) to characterize interventions delivered. Providers in regions randomized to continue EBI will continue service as usual.

#### Caregiver participation

Most CBAs include caregivers in some way because caregiver involvement is required by most funders. Providers in the EBI group will work with parents as usual. Providers in the ESDM condition will receive training in ESDM caregiver coaching strategies and will be asked to conduct caregiver coaching in the strategies at least monthly. Providers in the ESDM group will have training in use of “Help is in Your Hands” (HIIYH; www.helpisinyourhands.org), an online program for parents that includes 4 modules focused on video examples of families using the strategies during daily routines. Modules cover: (1) Increasing Children’s Attention to People; (2) Increasing Children’s Communication; (3) Creating Joint Activity Routines; (4) ABCs of learning. HIIYH includes the core elements of ESDM which align with the 11 essential common elements shared across NDBIs.

#### CBA provider training

Working with our CBA partners, we determined that the best training approach for this trial would be using our experienced ESDM trainers to train CBA supervisors using a combination of synchronous and asynchronous methods. Trainer fidelity to the training model will be tracked. Technicians will receive asynchronous didactic trainings combined with coaching and feedback from their CBA supervisors (who will receive support from the project ESDM team).

#### Supervisor training

Training will begin with a series of asynchronous, interactive (e.g., quizzes and activities), web-based lessons, followed by online coaching of supervisors by the project team through video review of their ESDM implementation and both technician and caregiver coaching. Supervisors will be trained to fidelity in all aspects of the ESDM model: assessment, goal development, data collection and intervention strategies. They will be trained to fidelity in ESDM coaching strategies to be used with both caregivers and technicians. Supervisors will use on-line ESDM parent training videos, Help is in Your Hands (HIIYH), and the ESDM caregiver manual [86] for caregiver coaching. After reaching ESDM fidelity with their trainer, supervisors will attend several web-based monthly peer supervision meetings with other participating supervisors that will include ongoing fidelity checks, to assure their continued development of ESDM delivery skills (see Table 1).

**Table 1:** Structure of the Early Start Denver Model (ESDM) Training Plan

Overview	Content & Format	Hours
ESDM Training for Supervisors		
What is the Early Start Denver Model	Introduction to ESDM theory and strategies, curriculum checklist, goal development, data collection. Format: asynchronous, interactive, video examples	2.5
ESDM Techniques and Strategies	Integrating joint activity routines. How to design and conduct an ESDM session; joint activity routines Format: asynchronous, interactive, video examples	2.5
Implementing Joint Activity Routines	Provider practices ESDM strategies (following child interest; sensitivity, themes etc.). Format: synchronous group video review	2^a^
Developing intervention plans	Using the ESDM Curriculum checklist for assessing learning strengths and needs. Using the checklist to create an intervention plan	3
Practice assessment and plan development	Practice conducting and coding curriculum checklist with feedback and developing goals until fidelity is met. Format: group video review, document review	2^a^
ESDM Coaching	Coaching during ESDM sessions until ESDM Fidelity is met. Format: Synchronous or video review	2^a^
Adult learning and providing coaching	Adult learning strategies to support successful coaching of technicians and caregivers in ESDM. Format: asynchronous	1
Practice with technician coaching	Using strategies to coach technicians	1^a^
Caregiver Coaching	Introduction to caregiver coaching strategies incorporating Help is in Your Hands Modules. Session preparation; collaborative coaching Format: asynchronous, interactive, video examples	7
Caregiver Coaching Feedback	Review of goal development and documentation; fidelity; Review of provider coaching caregiver sessions with ESDM trainer until fidelity is met. Format: Group video review	12^a^
Monthly Learning Collaborative	ESDM trainers host monthly learning collaborative for supervisors; Participants will provide case presentation, code ESDM fidelity and discuss challenges and successes	1 mo
ESDM Training for Technicians		
ESDM Introduction for Practice	Introduction to ESDM strategies and data collection. Format is asynchronous and includes interactive components and video examples	2
ESDM Intervention Training	Technicians will receive coaching and feedback from supervisors in accordance with the timing of training at their agency	varies
Just-in-Time (JIT) Micro Trainings	Access to JIT video modules (2–5 min) featuring ESDM examples matching age and goals of their current children. Modules can be viewed prior to treatment sessions to increase fidelity. Supervisors will assign at least 1 module per week for the first 8 weeks of training	varies

^a^additional coaching provided as needed to meet fidelity

#### Technician training

Technicians will complete asynchronous didactic training that includes an introduction to ESDM principles, strategies, and data collection. Supervisors will coach them in use of ESDM strategies using their agencies’ supervision model. Technicians will also view just-in-time (JIT) microlearning modules: specific 3 to 5-min lessons featuring a child of similar age, skill level and goals, just prior to an intervention session. Using JIT microlearning is an effective way to teach complex strategies [87, 88]. JIT learning provides immediate information when it is needed by delivering content in manageable units that fit technicians’ clinical schedules. Each JIT microlearning provides ideas for learning activities to teach a specific goal and brief information about how autistic children learn. A library of JIT videos will be made available and assigned to technicians by their supervisor. See Table 1 for the technician training plan.

#### Training materials

Supervisors will receive three ESDM manuals: the core treatment manual [72], a manual written for caregivers [86], and a manual on coaching caregivers in ESDM [86]. They will also receive access to HIIYH videos, caregiver coaching materials, a fidelity checklist for technicians, access to an ESDM goal bank, data collection tools, and access to JIT modules.

#### Fidelity to the ESDM training model

To assess fidelity to the ESDM training model, we will measure three training variables: (1) supervisor and technician completion of online training modules, JIT modules, and training activities will be tracked via the web-based training system; (2) supervisor participation in coaching and supervision activities, including receiving feedback and fidelity ratings from project staff for the curriculum assessment administration and scoring, goal development, ESDM implementation, caregiver coaching and technician coaching; and (3) trainer ESDM fidelity scores based on 25% of their ESDM Trainer coaching and supervision sessions coded by project staff. Supervisors who do not meet fidelity standards will receive supervision until they meet fidelity standards.

#### Treatment fidelity measures

We will assess supervisor ESDM fidelity at multiple levels: child skill assessment and goal development, ESDM strategy use, data practices, and coaching others. Supervisors and technicians will be coded on ESDM Strategy Use. Scoring sheets and the fidelity measures are available from the first author.

#### ESDM progress tracking and goal development

Supervisors will be scored on assessment and goal fidelity (curriculum checklist described below) on the ESDM Certification Rating System (CRS). Once using ESDM, they will submit curriculum checklists and objectives for each child enrolled in the study.

#### Caregiver and technician coaching

A modified version of the Coaching Practices Rating Scale (CPRS) [89] will evaluate supervisors’ fidelity to coaching strategies. Supervisors in both groups will submit one caregiver session and one technician supervision video per month for the duration of the study to examine fidelity. Each of the 13 fidelity items will be rated on a binary scale of present or absent, and these scores will be summed for a total of 13 possible points. Intraclass correlation coefficients in prior studies indicated high reliability: ICC = 0.92 (CI: 0.71–0.98).

#### ESDM strategy use fidelity

The ESDM Fidelity Checklist [72] will assess use of ESDM practices. The ESDM Fidelity Checklist consists of 13 items: (a) management of child attention; (b) ABC teaching format; (c) instructional techniques; (d) modulating child affect/arousal; (e) management of unwanted behavior; (f) use of turn-taking/dyadic engagement; (g) child motivation is optimized; (h) adult use of positive affect; (i) adult sensitivity and responsivity; (j) multiple varied communicative functions; (k) adult language; (l) joint activity and elaboration; and (m) transition between activities.

#### Use of NDBI strategies

To understand treatment differentiation between the ESDM and EBI groups we will code the use of NDBI strategies across groups. To examine differentiation between the interventions in a more valid and unbiased manner than simply using ESDM codes across conditions we will use the eight-item NDBI-Fi measure [90] developed to capture common elements across NDBI interventions. This measure has adequate reliability, sensitivity to change, and concurrent, convergent, and discriminative validity. We will use the total score and examine differences by strategy type, responsiveness, and directives, consistent with recent studies [91].

#### Use of Discrete Trial Teaching (DTT) strategies

To understand the quality of intervention in the EBI condition we will use a fidelity tool from Rogers et al., 2021 to measure correct implementation of typical EBI teaching using discrete trial strategies. The fidelity tool measures the correct implementation of 9 components using a 5-point Likert scale applied to randomly selected 20-min sections of recorded treatment sessions (Yoder P, McEachin J, Wallace E, Leaf R, 2014, unpublished). Discrete Trial Training Fidelity of Treatment Rating). During instruction, children typically have blocks of teaching trials interspersed with short breaks that include therapist interactions. Treatment blocks will be coded with the DTT and NDBI tools. Breaks will be coded using the NDBI tool.

Providers will upload intervention and coaching videos throughout participation in the study which will be coded for the above fidelity by trained research team members naïve to study arm.

### Procedures and measures

Child and family level outcomes will be assessed at three time points by trained assessors naïve to intervention condition: Baseline (BL), 6 months, and 12 months post BL. Outcome data will be collected by administering a brief battery of measures via distance technology that includes interview and survey and assessments with caregivers and video recordings. All assessors will be experienced MA or PhD level clinicians and supervised by a licensed clinical psychologist with over 20 years of experience in assessing young autistic children. All data will be entered directly into secure computer systems. Interviewers and video coders will be naive to group status (ESDM or EBI). The primary outcome will be child social communication and language (caregiver report and observational coding). Secondary outcomes are: (1) adaptive behavior and cognitive gains, (2) progress toward goals; (3) quality of life; (3) caregiver use of NDBI strategies; (4) increases in caregiver competence. We will assess engagement of the identified treatment mechanisms: child social motivation and caregiver use of NDBI strategies. Measures, constructs, and timing are listed in Table 2. Commonly used measures are described briefly. Newer or less standard measures are described in more detail.

**Table 2:** Assessments

Domain	Measure and description	Method	Timing		
			T1	T2	T3
**Characterization Measures**					
Demographics	Family Demographic Questionnaire	Caregiver Survey	X		
Devel Level	Devel. Profile -4th Ed, Cognitive	Caregiver Survey	X		
Treatment Type and Intensity	Ongoing Services Survey	Caregiver Interview	X	X	X
**Primary Outcomes**					
Language & Social Communication	Vineland Adaptive Behavior Scales-3 Communication Subscale	Caregiver Interview	X	X	X
	APPL	Direct Observation	X	X	X
**Secondary Outcomes**					
Adaptive Behavior	Vineland Adaptive Behavior Scales-3	Caregiver Interview	X	X	X
Quality of Life	CarerQoL; PEDSQL	Caregiver Survey	X	X	X
Caregiver Fidelity	NDBI-Fi	Direct Observation	X	X	X
Social Communication	Brief Observation of Social Change (BOSCC)	Direct Observation	X	X	X
Early Intervention Support and Processes	Family Outcomes Survey-Revised; Measure of Processes of Care	Self-Report		X	X
Potential Intervention Side Effects	Emotion Dysregulation Inventory-Young Children (EDI-YC) short form	Caregiver Survey	X	X	X
**Treatment Mechanisms**					
Social Motivation	PDDBI Social Approach Scale	Caregiver Survey	X	X	X
	JERI	Direct Observation	X	X	X
Caregiver Fidelity	NDBI-Fi	Direct Observation	X	X	X

T1 = baseline; T2 = 6 months; T3 = 12 months

Participant retention will be facilitated by frequent contact with the research team, gift cards for measure completion, birthday cards sent to children and assessment reports. If child participants leave the agency we will still attempt to obtain measures at each timepoint.

### Characterization measures

#### Treatment type and intensity

Caregivers will complete an interview regarding intervention services received during the study period. In addition, we will track the number of CBA-provided treatment hours and caregiver coaching attendance via agency records.

#### Cognitive level

The Developmental Profile-4 (DP-4) [92]. Cognitive Scale is a standardized caregiver interview measure that produces norm-referenced scores for the cognitive domain. Test–retest reliability for the Cognitive scale is 0.83; internal consistency 0.82 to 0.94. Construct validity was verified with comparison of established measures (cognitive scale = 0.57).

#### Primary child outcomes: social communication and language

We will examine the effect of ESDM training on children’s social-communication and language using observational coding and caregiver report.

The Assessment of Phase of Preschool Language (APPL) [93] operationalizes research-based language development stages [94]. Language phases are derived from spoken language or augmentative communication systems and standardized assessments. The APPL characterizes expressive language domains: phonology, vocabulary, grammar, and pragmatics. For each domain, the APPL outlines the range of demonstrated skills that could meet criteria for each phase: Phase 1: Preverbal; Phase 2: First Words; Phase 3: Word Combinations, Phase 4: Sentences, or Phase 5: Complex Language. The APPL has strong interrater reliability and good construct validity A Language samples will be obtained from transcriptions of child-caregiver interactions recorded at each timepoint (see video collection). The APPL has been used to examine change in language level in multiple autism studies.

#### Vineland communication domain

The Vineland Adaptive Behavior Scales-3 (VABS-3) [95] consists of four domains of adaptive behavior: communication, daily living skills, socialization, and motor skills. It has been validated with children with developmental disabilities The scales yield normative standard scores (M = 100; SD = 15) that can be used for comparison across groups. The communication domain will be used to examine overall change in communication in the natural environment. The Vineland Interview edition will be used to obtain parent report of adaptive skills.

#### Secondary outcomes

##### Adaptive behavior

*Vineland Adaptive Behavior Scales-3* (VABS-3) [95] domains of adaptive behavior: daily living skills, socialization, and motor skills will be examined as secondary outcomes.

##### Caregiver and child quality of life

The CarerQoL [96]assesses perceived caregiver quality of life across seven dimensions. The Pediatric Quality of Life Inventory (PedsQL) [97] assesses children’s quality of life across four domains based on caregiver report and has been validated in an autism population [98].

##### Brief observation of social communication change

(BOSCC; [99]. The BOSCC consists of 15 items coded based on video observations on a 6-point scale ranging from 0 (the characteristic is not present) to 5 (the characteristic is present and it significantly impairs functioning). Thus, higher scores indicate more autism characteristics. Items 1–8 focus on Social Communication (SC), while items 9–12 capture Restricted and Repetitive Behaviors (RRBs). The BOSCC results in SC (i.e., eye contact, facial expressions, gestures, vocalizations, integration of vocal and non-vocal communication, frequency/function of social overtures, frequency/quality of social responses, engagement in activities/interaction, and play with objects) and RRB domain totals (unusual sensory interests, hand/finger or other complex mannerisms, and unusually repetitive interests/stereotyped behaviors). The Core total combines the SC and RRB scores. We will not be targeting autistic characteristics in our project. We will include the BOSCC as a secondary measure of social communication to facilitate comparison across studies.

##### Early intervention support and processes

The *Family Outcomes Survey-Revised* (FOS-R) [100] is a 41-item measure uses a 5-item Likert scale to assess parents’ perceived strengths and needs as they relate to the early intervention support they receive. The FOS-R has good internal consistency in English (subscales ranging from 0.73 to0.95 for Cronbach’s alpha). [100] The *Measure of Processes of Care—20* (MPOC—20) [101] measures how family-centered parents perceive their child’s intervention services. The 20-item scale asks parents to rate how much people who work with their child (a) enable partnership, (b) provide general information, (c) provide specific information about their child, (d) coordinate comprehensive care for the child and family, and (e) are respectful and supportive. The scale has good internal consistency with coefficients ranging from 0.83 to 0.90 [101].

##### Intervention side effects & harm

*Emotion Dysregulation Inventory-Young Children* (EDI-YC) [102] short form measures emotion dysregulation with two scales, reactivity and dysphoria. Reactivity is characterized by rapidly escalating, intense, labile negative affect and difficulty downregulating that affect. Dysphoria is characterized by poor up regulation of positive emotion. This 14-item scale has been used with children on the autism spectrum. The measure has good validity and is supported by expert review. If children have > 1sd of change in this measure over time or providers or parents report regression the research team and data safety and monitoring board will assess for discontinuing or modifying the intervention and/or study participation.

#### Treatment mechanisms variables

##### Social motivation and caregiver NDBI fidelity

Social motivation will be measured in two ways and those assessments will be used to examine proximal and distal changes to the intervention mechanism and its role as a moderator.

*Pervasive Developmental Disorder Behavior Inventory (PDDBI)* [103] examines characteristics of autism in children between 18 months and 12.5 years through caregiver reports. It has high internal consistency (0.84—0.97), inter-rater reliability (ICC = 0.75—0.93), and good construct validity. The Social Approach subscale will provide a distal measures of social motivation (treatment mechanism) and includes 36 items representing all three behavioral manifestations of social motivation. Studies using the Social Approach subscale report good consistency (α = 0.94) and test–retest reliability of 0.93 [104, 105].

Joint Engagement Rating Inventory (JERI) [106] will be a proximal, objectively rated measure of child social motivation during adult/child interactions. The JERI is widely used to examine child behavior in autism studies and has high validity and reliability. One score per code will be assigned to each Communication Play Protocol observation (see below) and averaged across the 3 activities for analyses.

##### Caregiver NDBI fidelity

Caregiver-child interaction videos (see below) will be coded using the NDBI-Fi Checklist (see Fidelity measures and video collection).

#### Video data collection

Video data will be collected for outcomes at three time points using the Communication Play Protocol (CPP) [107]. The CPP produces video records of three 5-min semi-structured scenes that focus on requesting, social interacting, and shared commenting. We will collect two CPP videos at each time point, one with the caregiver and one with a provider that does not know the child and is naïve to condition. Video data will be coded using the (1) APPL for children language outcomes; (2) JERI to assess social motivation; and (3) and NDBI-Fi to examine caregiver use of ESDM/NDBI strategies.

##### Video coding procedures

Trained coders naïve to group, timepoint, and study aims will code video measures to avoid bias. Each coder will be trained in one scoring system to reliability (80% agreement over 3 videos). For each measure, a random sample of 20% of sessions will be double coded for inter-rater reliability throughout coding. If agreement drops below 80%, training will be provided until agreement is achieved.

#### Analytic plan

In this trial, there are several levels of clustering: repeated observations are nested within the child/caregiver, the child/caregiver is nested within the team, teams are nested within the region, and regions are nested within CBAs. Therefore, we will use a modeling strategy that includes random intercepts for region and/or CBA, team, and random child/caregiver effects (intercept, slopes, as appropriate). All primary analyses will be conducted on an intent-to-treat basis using a generalized linear mixed-effects models framework [103], which can accommodate continuous, binary, and count outcomes through an appropriate choice of link function. Preliminary analyses will involve examining the outcomes and covariates to verify their appropriateness, identifying patterns of missing data, and conducting a multivariate outlier analysis. Model validation will be carried out using both analytical and graphical techniques to check core assumptions such as linearity, distribution, and homoscedasticity. Transformations of outcome variables will be considered if suggested by the model validation analyses. All analyses will include available relevant biological variables, including child or caregiver sex and age and baseline characteristics if there is any evidence of randomization imbalance in them. Randomization should produce intervention and control groups that are comparable and balanced. As a first-order check on confounding, we will examine the success of randomization by comparing baseline characteristics of children, caregivers, and providers assigned to the two study arms. Where clinically significant differences are apparent, child-, caregiver-, and provider-specific covariates will be added to the statistical models as fixed predictors to examine whether the intervention effect is robust in their presence.

#### Primary and secondary outcomes

The analytic approach for each primary and secondary outcome measure will follow the same general model-building strategy. For outcomes assessed at baseline, 6 months, and 12 months, the models will include fixed effects for time (baseline, 6-, and 12-months), group, and their interaction, as well as covariates (e.g., child/caregiver sex, age, etc.) and random effects for child/caregiver, team, or region to account for clustering. The interaction between time and group will directly test the hypothesis that participants in the ESDM group show greater improvement than those in the EBI group. In all models, we will consider adding relevant covariates related to child/caregiver or provider-level characteristics if randomization at the region level did not ensure comparability between the two groups [108].

#### Moderation analyses

Moderation analyses will explore the differential effectiveness of the two interventions by maternal level of education (as a proxy for SES), child race/ethnicity, and technician ESDM fidelity. We will build upon the primary models with treatment group by time effects by incorporating interaction terms for moderators of interest and conducting sub-group analyses. For each target moderator (e.g., maternal education), we will add the 3-way treatment group by time by moderator interaction term (and all lower-order 2-way and main effects) to determine whether differences between treatment groups in change over time for a given outcome variable are modified by target moderators. A significant 3-way interaction effect will indicate the presence of treatment effect heterogeneity between subgroups. Following this, we will conduct simple effect analysis to estimate treatment effect differences (i.e., difference in changes over time between arms) within each subgroup. For adherence to ESDM fidelity at the technician level, we expect substantial differences between treatment groups, and plan to investigate this as a moderator of all child outcomes.

#### Mediation analyses

Conceptually, social motivation (measured by PDDBI and JERI) and caregiver NDBI fidelity can be viewed as an intermediated outcome (mediator, M). The intervention may affect the primary outcomes indirectly through a pathway of the mediator (M). To test the mediated effect (or mechanism of change), we will conduct a mediation analysis by extending the generalized mixed effects models specified for assessing treatment group differences in primary outcome variables by adding continuous ratings of social motivation and parent fidelity, respectively, as predictors to the model describing language improvement. The mediation analysis will follow a standard series of steps: (1) Test for the direct effect of the treatment group on the primary outcome variable as represented by the time by treatment group interaction effects (i.e., the primary model); (2) Test for the time by treatment group interaction effect using the measure of social motivation (or parent fidelity, respectively) as the dependent variable in an analogous mixed effects model to assess the coefficient for group differences in change over time in the target mediator; (3) Return to the model in step 1 and add the main effect of time-varying social motivation scores (or parent fidelity, respectively) in predicting outcome scores to assess the direct relationship between the target mediator and outcome while controlling for the time by treatment group effect on outcome; (4) Calculate the degree and significance of the indirect effect using Monte Carlo simulations of the estimated coefficients and their respective standard errors.

#### Missing data

Our protocols include numerous provisions to minimize the amount of missing data, and our team has achieved high retention rates in previous work. However, some data will inevitably be missing. We will use standard methods to evaluate missing data assumptions and to determine alternative analytic strategies if needed. One of three approaches will be used: First, if the proportion of missing data is small and there is evidence that data are missing at random (MAR), all available data will be analyzed using the maximum-likelihood estimation procedures described above. Second, if the proportion of missing data is nontrivial with evidence that data are MAR, multiple imputations for repeated measurements will be used to generate complete data. Third, if there is evidence of a non-MAR mechanism for missing data, pattern mixture models will be used to evaluate and control for the missing data pattern.

#### Power considerations

Given that the proposed analyses for primary outcomes will employ mixed effects modeling of clustered data to assess differences in changes from baseline between treatment groups, power analyses were conducted using Monte Carlo simulations of multi-level models in SAS (SAS Institute Inc., Cary, NC). Expected fixed effect values for effects of interest (e.g., treatment group by time interactions) were obtained from prior research on ESDM treatments and developmental change [109]. We assumed a range of plausible intraclass correlation coefficient (ICC) values for the random effects of child/caregiver dyad (0.3 to 0.5), team (0.1 to 0.25), and region (0.05 to 0.1) based on previous community intervention studies and pilot data and accounted for a 10% dropout rate. We used a type I error level of 5%.

Under each scenario, our proposed sample size of 300 children/caregivers, 100 teams, and 20 centers would provide at least 80% power to detect a standardized improvement in children’s social communication and language of *d* = 0.6. We assumed that 10% of children would not contribute any data; however, the participants who dropped out would have provided some data and will contribute to the analyses. Therefore, our calculations are conservative.

#### Procedures and measures addressing our exploratory implementation aim

We will measure implementation during the three EPIS phases: adoption (recruitment), implementation (ESDM training and delivery), and predicted sustainment (after the research study). We will measure acceptability, appropriateness, and feasibility of the intervention, and provider, family, and organizational characteristics to identify determinants of ESDM implementation. We will use a combination of surveys and structured interviews (see Table 3).

**Table 3:** Implementation Measures

Construct	Measure/Indicator
Adoption	Decision to participate in effectiveness study (yes/no)
Implementation	ESDM provider adherence measures [ESDM Fidelity; NDBI Fidelity]; Adaptations to Evidence-Based Practice Scale
Sustainment	Ongoing use of ESDM [PRESS; interviews with managers and providers]
ESDM Appropriateness, Feasibility, Acceptability	Perceived fit of ESDM with agency, provider, and family [AIM; interviews] Caregiver engagement in intervention [session attendance]
Provider Characteristics	Perceived self-efficacy [Autism Self-Efficacy Scale] Provide background experience, previous EBP training
Organization Characteristics	Financing structure and reimbursement; # autistic clients under age 5; Implementation Climate Survey

Acceptability of Intervention Measure (AIM), Intervention Appropriateness Measure (IAM), & Feasibility of Intervention Measure (FIM) [110] determine the extent to which a participant believes an intervention is acceptable, appropriate, and feasible and have strong internal consistency (AIM α = 0.89; IAM α = 0.87; FIM α = 0.89). All participating providers and caregivers will complete these scales every six months during participation. Total score on each scale will be used.

Adaptations to Evidence-Based Practices Scale (AES) [111] is a 6-item scale assessing provider adaptations to EBPs delivered. Providers rate six items using a 5-point Likert scale (0 “not at all,” 4 “a very great extent”) to indicate the extent to which they made each type of adaptation when delivering a specified EBP, including (a) modifying the presentation of EBP strategies, (b) shortening or condensing the pacing of the EBP, (c) lengthening or extending the pacing of the EBP, (d) integrating supplemental content or strategies, (e) removing or skipping components, and (d) adjusting the order of sessions or components.

Provider Report of Sustainment Scale (PRESS) [112] captures provider report of continued use of an intervention. The PRESS has good psychometric properties across multiple interventions and service systems and strong construct validity.

Autism Self-Efficacy Scale for Teachers (ASSET) [113] is a 30-item self-report measure of providers’ beliefs about their ability to implement appropriate teaching strategies when working with autistic children. We adapted the measure for use with community providers who rate their efficacy in carrying out several different assessment, intervention, and evidence-based practices relevant to autism early intervention. Providers rate their self-efficacy using a scale from 0 (*cannot do at all*) to 100 (*highly certain can do*). The total score is calculated as the mean score across the 30 items. Scale internal consistency is 0.96.

The Implementation Climate Scale (ICS) [114, 115] measures employees’ shared perceptions of the policies, practices, procedures, and behaviors that are expected, rewarded, and supported to facilitate effective EBI implementation. The ICS has good psychometric properties across several settings including good internal consistency and good construct validity.

Implementation Interview: Semi-structured interviews will be conducted with regional managers, supervisors, technicians, and caregivers to gather additional information on ESDM feasibility, usability, acceptability, fit (including cultural fit with family needs) and plans for sustainment. We will conduct interviews with a subset of participants until we reach saturation (approximately 30 in each group). Facilitators will follow a semi-structured interview guide [116, 117].

#### Data analysis (exploratory)

Descriptive data regarding feasibility, acceptability and fit and qualitative interview data will be examined every 6 months. These data will be used iteratively through the implementation phase of the trial to make culturally relevant adaptations to the intervention. Adaptations will be carefully logged and tracked and resulting outcomes monitored using recommended methods. We will explore descriptive statistics for the various measures of organizational and provider characteristics and participation and will use predictive models (with multi-level modeling as above) to understand appropriateness, feasibility, and acceptability in the ESDM treatment group. Given that measures of implementation (e.g., provider fidelity) are important for understanding the feasibility of scaling ESDM to CBAs, we will analyze such implementation measures as dependent variables and examine other variables in Table 3 (e.g., organization characteristics, perceived fit) as predictors of individual provider variability in fidelity.

Qualitative Data analysis. NVivo QSR 11 [118] will be used for qualitative analyses. A framework-driven analytic approach will guide the coding process [44, 119]. Coders will use an iterative coding and review process informed by grounded theory [120].

#### Integration of qualitative and quantitative analyses

A sequential Quan > QUAL mixed method design will be employed [121]. The primary functions of the mixed-methods analyses will be convergence and expansion.

## Discussion

This project is one of the first large-scale, randomized hybrid effectiveness trials of an autism early intervention. The large and diverse sample will allow us to examine how well ESDM conducted by CBAs activates the hypothesized mechanism of the intervention, social motivation. Understanding how social motivation works to determine response to NDBI will allow for improvement of intervention strategies that enhance social motivation to facilitate improved outcomes. Examining the role of social motivation in a diverse community sample of toddlers and young children on the autism spectrum will inform the social motivation hypothesis of autism, which, to date, has only been tested in research samples. Additionally, the relationship between caregiver fidelity and child outcomes has not been examined in a large, diverse sample that may have varying cultural values regarding intervention delivery.

The impact of the study is likely to be greater because the project utilizes partnerships with family members, autistic adults, and CBAs to develop both the proposal and to carry out the project, assuring relevance of the research questions to the community and increase the potential for uptake of the intervention [122]. Our community partners assure us that this adapted ESDM training and intervention model fits with the current CBA intervention and financing structure. By engaging an extensive network of CBAs supporting autistic children as the intervention deliverers, positive findings can readily generalize to other CBAs and increase access to diverse regions and children.

The proposal is responsive to the neurodiversity perspective. When used correctly, ESDM builds in respect for children’s interests and preferences with a strengths-based approach. ESDM emphasizes the importance of responsive, sensitive relationships between adults and children and on outcomes associated with development, quality of life, and adaptation instead of reduction of unwanted behaviors and “normalization”. The developmentally and culturally appropriate naturalistic interactions of ESDM have the potential to increase the acceptability of early intervention and increase adoption by families concerned about the long-term effects of ABA on their child’s emotional well-being.

The effectiveness trial methodology will be harnessed to examine implementation determinants to facilitate scale up. Hybrid Type 1 trials allow implementation determinants to be identified more comprehensively and earlier than in a sequential model [123]. This study uses an established implementation framework, EPIS, to facilitate effective prospective design to support future scale-up studies with diverse populations. Implementation data can be used iteratively through the implementation phase of the trial to make culturally relevant adaptations to the intervention. Adaptations, which will be carefully logged and tracked, and resulting outcomes monitored using recommended methods [124] will increase the scalability of the intervention.

Results of the project will be disseminated to several different audiences using methods specifically designed to reach each of them. Target audiences are community-based agencies, researchers and their students, state and community program administrators and providers, and parents and funders. A project website will describe the project and provide tools and information for all audiences and a method for requesting more information about the study for interested community agencies. The website will include infographics and lay abstracts of study presentation and publications. Data will be presented through conference presentations, social media, journal articles, lay publications and presentations to policy makers and funders. All publications will be made publicly available through the University of California and PubMed Central.

## Data Availability

Data will be available through the NIMH Data Archive. Descriptive/raw data will be submitted semi-annually, and additional data will be submitted at the time of publication. In addition, all datasets developed for the study will be available from the corresponding author upon reasonable request.

## Abbreviations

CBA: Community Based Agency
EBI: Early Behavioral Intervention
ESDM: Early Start Denver Model
NDBI: Naturalistic Developmental Behavior Intervention
